# Clinical efficacy of abatacept, tocilizumab, and etanercept in Japanese rheumatoid arthritis patients with inadequate response to anti-TNF monoclonal antibodies

**DOI:** 10.1007/s10067-014-2711-2

**Published:** 2014-06-28

**Authors:** Shinya Hirabara, Nobunori Takahashi, Naoki Fukaya, Hiroyuki Miyake, Yuichiro Yabe, Atsushi Kaneko, Takayasu Ito, Takeshi Oguchi, Daihei Kida, Yuji Hirano, Takayoshi Fujibayashi, Fumiaki Sugiura, Masatoshi Hayashi, Koji Funahashi, Masahiro Hanabayashi, Shuji Asai, Naoki Ishiguro, Toshihisa Kojima

**Affiliations:** 1 Department of Orthopedic Surgery and Rheumatology, Nagoya University Hospital, Nagoya University Graduate School of Medicine, 65 Tsuruma-cho, Showa-ku, Nagoya, Aichi Japan; 2 Department of Rheumatology, Toyohashi Municipal Hospital, 50 Hachiken-nishi, Aotake-cho, Toyohashi, Japan; 3 Department of Orthopedic Surgery, Kariya-Toyota General Hospital, 5-15 Sumiyoshi-cho, Kariya, Japan; 4 Department of Orthopedic Surgery, Ichinomiya Municipal Hospital, 2-2-22 Bunkyo, Ichinomiya, Japan; 5 Department of Rheumatology, Tokyo Shinjuku Medical Center, Tsukudo-cho, Shinjuku-ku, Tokyo Japan; 6 Department of Orthopedic Surgery and Rheumatology, Nagoya Medical Center, 4-1-1 Sanno-maru, Naka-ku, Nagoya, Aichi Japan; 7 Ito Orthopedic Clinic, 3-812 Ueda-minami, Tenpaku-ku, Nagoya, Aichi Japan; 8 Department of Orthopedic Surgery, Anjo Kosei Hospital, 28 Higashihirokute, Anjo, Japan; 9 Department of Orthopedic Surgery, Konan Kosei Hospital, 137 Oomatsubara, Takaya-cho, Konan, Aichi, Japan; 10 Department of Rheumatology, Nagano Red Cross Hospital, 5-22-1 Wakazato, Nagano, Japan

**Keywords:** Abatacept, Etanercept, Rheumatoid arthritis, Switching medications, Tocilizumab

## Abstract

The aim of this study was to compare the efficacy and retention rates of three biologics (abatacept, tocilizumab, and etanercept) after switching from first-course anti-TNF monoclonal antibody therapy. We performed a retrospective multicenter study of 89 patients who underwent second-course biologic therapy for 52 weeks after switching from first-course anti-TNF monoclonal antibody therapy. Patients at baseline had a mean age of 58.7 years, mean disease duration of 9.8 years, and mean clinical disease activity index (CDAI) of 22.4. There was no significant difference between the three drugs, except in rheumatoid factor positivity. Retention rates for abatacept, tocilizumab, and etanercept treatment at 52 weeks were 72.0, 89.5 and 84.6 %, respectively. The evaluation of CDAI indicated no significant difference at 52 weeks among the three drugs. Discontinuation due to all unfavorable causes did not significantly differ among the three drugs in hazard ratio-based evaluations. Our results show that patients treated with abatacept, tocilizumab, and etanercept achieved a high response rate with no significant differences in drug retention rates and clinical efficacy. These drugs represent good therapeutic options for patients with RA who are refractory to anti-TNF monoclonal antibody therapy.

## Introduction

Rheumatoid arthritis (RA) is a chronic and systemic autoimmune inflammatory disease that clinically manifests as joint pain and swelling [1]. In the past decade, treatment of RA has improved significantly with the introduction of tumor necrosis factor inhibitors (TNFi), which reportedly demonstrate high efficacy [2–4]. However, these drugs have little or no effect in about 30 % of treated patients, with two thirds demonstrating moderate to high disease activity at 1 year post-treatment [5]. In clinical practice, switching biologics remains a difficult issue. According to the European League Against Rheumatism recommendations, patients who do not respond to initial TNFi therapy should switch to a different TNFi or use a different class of biologics (abatacept, rituximab, or tocilizumab) [6]. While some studies reported on the outcomes of switching from TNFi to other biologics [7, 5, 8], no consensus has been reached on the strategy of switching.

Loss of therapeutic efficacy is readily observed with anti-TNF monoclonal antibodies (adalimumab and infliximab) in patients receiving concomitant low-dose methotrexate (MTX) due to immunogenicity-related issues [9–11]. This is one factor leading to withdrawal from anti-TNF monoclonal antibody therapy. The dose of concomitant MTX in Japan is lower compared to other countries [12], and switching from anti-TNF monoclonal antibodies is often required. To this end, we compared three drugs (abatacept, tocilizumab, and etanercept) that are considered to exhibit low immunogenicity [13, 14, 2].

In this study, etanercept, a drug with proven efficacy, was compared with abatacept and tocilizumab. In view of the different characteristics of available TNFi, switching from an anti-TNF monoclonal antibody to a TNF receptor fusion protein (etanercept) may be helpful if initial treatment fails. On the other hand, several new biologics with different mechanisms of action are now available (e.g., abatacept, rituximab, and tocilizumab). Some reports have compared switching to tocilizumab and abatacept [15, 16, 7]. Hyrich et al. reported that when the first TNFi treatment fails, the best alternative is to start on a different class of biologics [16]. However, there are no reports to date comparing new biologics with etanercept. Accordingly, this study aimed to compare patients who switched to etanercept, abatacept, and tocilizumab from first-course anti-TNF monoclonal antibody therapy.

Abatacept and tocilizumab are recently approved non-TNFi biologics that are marketed for the treatment of RA. Abatacept is the first member of a new class of biologics which inhibit T-cell activation by binding to CD80/86 and modulating its interaction with CD28. Based on this mechanism, abatacept is expected to achieve clinical efficacy in patients who respond inadequately or are naïve to other classes of biologics. Tocilizumab, a humanized monoclonal antibody against the IL-6 receptor, was approved in 2008 for use in clinical practice in Japan. The efficacy of tocilizumab for RA has been demonstrated in several clinical trials [14, 17] as well as in actual practice [18, 19]. Both drugs show low immunogenicity, with anti-drug antibody production rate of 2.3 % for abatacept and 2.5 % for tocilizumab [13, 14]. The efficacy and safety of these drugs in patients who are naïve or refractory to TNFi therapy have been demonstrated in several randomized controlled clinical trials (RCTs) [20–23]. However, controversy exists as to whether a different TNFi (e.g., etanercept) should be selected or other elements of the inflammatory process should be modified when switching from anti-TNF monoclonal antibodies.

Patients may exhibit differential responses to the three agents (abatacept, tocilizumab, and etanercept) upon switching, although there is no direct evidence to support this. The present study compared retention rates and clinical efficacy of abatacept, tocilizumab, and etanercept switched from first-course anti-TNF monoclonal antibody therapy based on retrospectively registered observational data.

## Materials and methods

### Tsurumai Biologics Communication Registry

The Tsurumai Biologics Communication Registry (TBCR) was developed in 2008 to explore the long-term prognosis of biologics in clinical practice and consisted of patients who were starting biologic treatments. Data were collected prospectively from 2008 and retrospectively for patients treated up to 2008 [24]. The present study included all patients (*n* = 89) who switched to abatacept, tocilizumab, or etanercept as a second biologic agent from first-course anti-TNF monoclonal antibody due to inadequate efficacy from September 2010 to September 2011 at Nagoya University Hospital or one of 12 other institutions affiliated with the TBCR and were prospectively enrolled in the TBCR. During the study period, we were able to choose freely among the five biological DMARDs (infliximab, etanercept, adalimumab, tocilizumab, abatacept) at our discretion as a second-line as well as a first-line biologic. All patients met the 1987 American College of Rheumatology classification criteria for RA and received abatacept, tocilizumab, or etanercept infusions according to the drug label and Japan College of Rheumatology guidelines for treatment. Patient anonymity was maintained during data collection, and the security of personal information was strictly controlled. This study was approved by the Ethics Committee of the Nagoya University Graduate School of Medicine.

### Data collection

Data were retrospectively collected from clinical records. The following demographic data were recorded at the initiation of treatment (baseline, week 0): disease duration, concomitant treatment (methotrexate [MTX] or prednisolone), joint damage (Steinbrocker stage), and daily dysfunction (Steinbrocker class). The following disease parameters were recorded at baseline and at 24 and 52 weeks of treatment: tender joint count (TJC) and swollen joint count (SJC) on 28 joints, general health on a visual analog scale (GH-VAS), and serum C-reactive protein (CRP) levels. Disease activity was evaluated at each time point using the 28-joint disease activity score with CRP (DAS28-CRP) and the clinical disease activity index (CDAI) which included data from the cited disease parameters.

### Statistical analysis

Demographic and disease characteristics were reported using descriptive statistics. All results are expressed as mean ± SD or percentage. Student’s *t*-test was used for two-group comparisons and the chi-square test for categorical variables. The last observation carried forward (LOCF) method was used in each analysis. All statistical tests were two-sided, and significance was defined as *p* < 0.05. Drug continuation rates were estimated by plotting Kaplan–Meier curves and were compared using log-rank test. Hazard ratios (HRs) for cause-specific drug discontinuation were calculated using the Cox proportional hazards model, adjusted for variables such as disease duration, age, sex, and concomitant use of MTX and CDAI. All analyses were performed with SPSS version 20.0.0 software (IBM Corp., Armonk, NY, USA).

## Results

### Patients

We examined 89 patients who switched to abatacept, tocilizumab, and etanercept as a second biologic agent from first-course anti-TNF monoclonal antibody therapy due to inadequate efficacy. Of these, 25 (28.1 %) had switched to abatacept, 38 (42.7 %) had switched to tocilizumab, and 26 (29.2 %) had switched to etanercept.

Baseline characteristics of all patients are shown in Table 1, categorized by the second biologic agent. Mean age was 58.7 ± 12.1 years, mean disease duration was 9.8 ± 8.3 years, and mean DAS28-CRP and CDAI were 4.6 ± 1.2 and 22.4 ± 11.0, respectively. A significant difference was found in rheumatoid factor positivity among the three drugs. No significant differences were found in factors reported to affect the effects of biologics, including MTX use, MTX dose, and disease duration. In the present study, the rate of concomitant MTX use was 78.7 %, with a mean dose of 7.4 mg/week.

**Table 1:** Baseline characteristics of patients with rheumatoid arthritis who switched from anti-TNF monoclonal antibodies

	Overall (*n* = 89)	Abatacept (*n* = 25)	Tocilizumab (*n* = 38)	Etanercept (*n* = 26)	*p* value
Age (year)	58.7 ± 12.1	62.8 ± 9.3	56.7 ± 12.4	57.5 ± 13.3	0.315
Sex (% female)	82	80	78.9	88.5	0.593
Disease duration (year)	9.8 ± 8.3	11.4 ± 9.5	7.9 ± 6.1	11.0 ± 9.6	0.207
Stage (I/II/III/IV, %)	19.1/21.3/24.7/32.6	20.0/20.0/24.0/36.0	19.4/22.2/33.3/25.0	19.2/23.1/15.4/42.3	0.627
Class (I/II/III/IV, %)	13.5/50.6/29.2/4.5	12.0/52.0/36.0/0	16.8/52.8/27.8/2.8	11.5/50.0/26.9/11.5	0.453
RF positive (%)	82.9	70.6	78.6	96	**0.002**
Previous biological DMARDs (%)					
Adalimumab	37.1	60	26.3	30.8	
Infliximab	62.9	40	73.7	69.2	
MTX use (%)	78.7	80	73.7	84.6	0.567
MTX dose (mg/week)^a^	7.4	7.5	7.3	7.8	0.716
Oral steroid use (%)	58.4	64	59.5	53.8	0.760
Oral steroid dose (mg/day)^a^	4.2	3.8	4	4.8	0.433
MMP-3 (ng/mL)	257.0 ± 235.2	217.1 ± 190.0	317.4 ± 271.6	183.9 ± 129.0	0.371
SJC, 0–28	5.4 ± 4.8	5.9 ± 5.6	5.7 ± 4.9	4.7 ± 3.7	0.546
TJC, 0–28	6.4 ± 5.6	5.3 ± 4.1	6.0 ± 5.7	7.9 ± 6.6	0.439
ESR (mm/h)	53.1 ± 27.1	57.4 ± 32.1	51.1 ± 24.9	53.0 ± 26.4	0.475
CRP (mg/dL)	2.6 ± 2.6	1.7 ± 1.9	2.9 ± 2.8	3.0 ± 2.9	0.374
GH-VAS 0–100 mm	54.1 ± 22.9	53.7 ± 25.2	53.9 ± 22.4	54.6 ± 22.3	0.514
DAS28-ESR	5.3 ± 1.2	5.2 ± 1.2	5.3 ± 1.2	5.4 ± 1.3	0.267
DAS28-CRP	4.6 ± 1.2	4.4 ± 1.1	4.7 ± 1.2	4.8 ± 1.1	0.266
CDAI	22.4 ± 11.0	21.2 ± 11.0	22.4 ± 11.1	23.5 ± 11.2	0.266
SDAI	24.8 ± 11.6	23.1 ± 11.3	24.7 ± 11.5	26.4 ± 12.3	0.335

Data are presented as mean ± SD, unless otherwise indicated

*Stage* Steinbrocker stage, *Class* Steinbrocker class, *RF* rheumatoid factor, *MTX* methotrexate, *MMP-3* matrix metalloproteinase-3, *SJC* swollen joint count, *TJC* tender joint count, *ESR* erythrocyte sedimentation rate, *CRP* C-reactive protein, *GH-VAS* general health visual analog scale, *DAS28* disease activity score in 28 joints, *CDAI* clinical disease activity index, *SDAI* simplified disease activity index

^a^Mean among patients receiving the drug

### Drug continuation rates

**Fig. 1:**
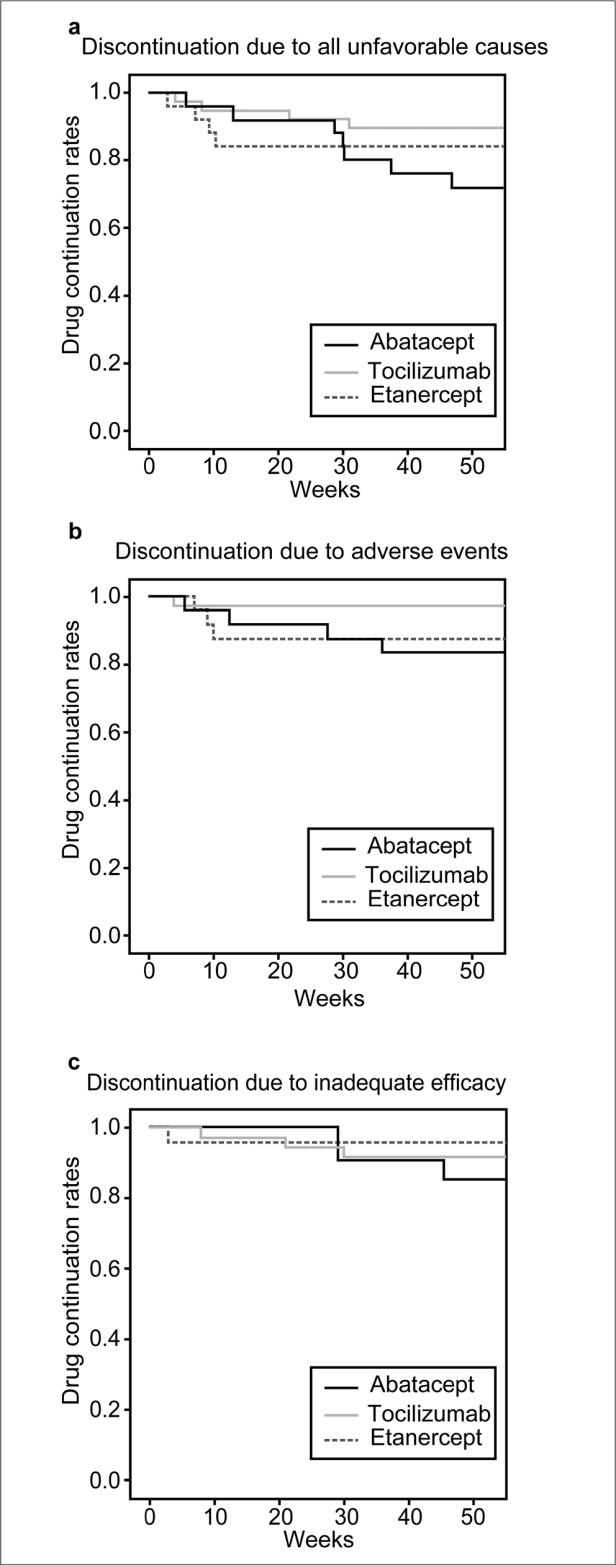
Patient retention in abatacept, tocilizumab, and etanercept treatment. Kaplan–Meier curves of treatment continuation rates among patients with rheumatoid arthritis over 52 weeks of treatment. **a** Discontinuation due to all unfavorable causes. **b** Discontinuation due to adverse events. **c** Discontinuation due to inadequate efficacy

Drug continuation rates were analyzed with Kaplan–Meier curves (Fig. 1). At 52 weeks, continuation rates for abatacept, tocilizumab, and etanercept were 72.0, 89.5, and 84.6 %, respectively (log-rank test, *p* = 0.121), for discontinuation due to all unfavorable causes (Fig. 1a). When classified according to reasons for discontinuation, continuation rates at 52 weeks for abatacept, tocilizumab, and etanercept were 88.0, 97.1, and 90.5 % (log-rank test, *p* = 0.374), respectively, for discontinuation due to adverse events (Fig. 1b), and 82.6, 91.9, and 95.7 % (log-rank test, *p* = 0.182), respectively, for discontinuation due to inadequate efficacy (Fig. 1c). It should be noted that discontinuation of tocilizumab due to adverse events and discontinuation of etanercept due to inadequate efficacy were low, although there was no significant difference. All drugs exhibited good retention rates.

### Clinical efficacy

**Fig. 2:**
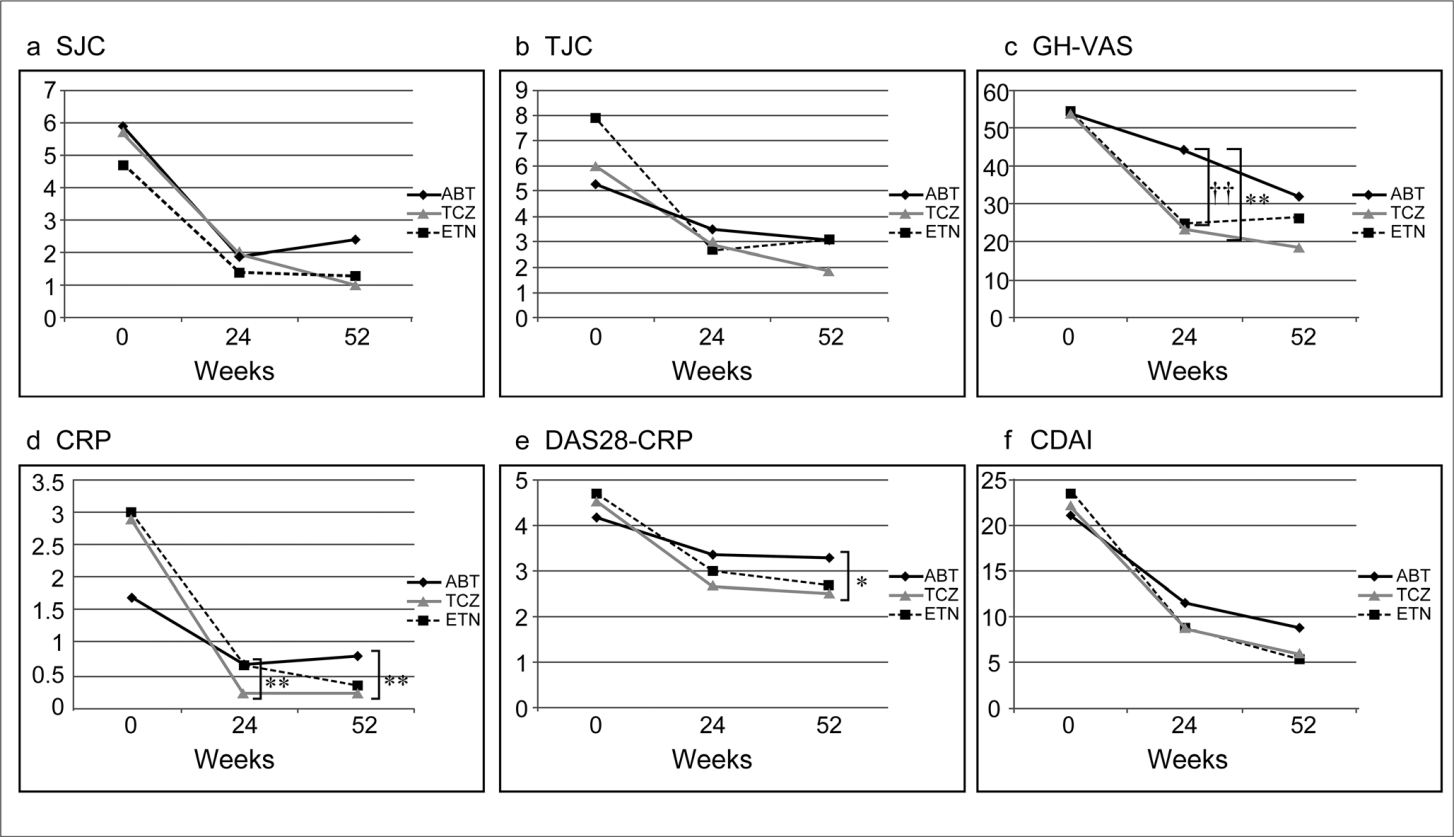
Overall clinical efficacy of switching biologics in patients with rheumatoid arthritis. Mean values for **a** swollen joint count (*SJC*), **b** tender joint count (*TJC*), **c** general health on a visual analog scale (*GH-VAS*), **d** C-reactive protein (CRP), **e** 28-joint disease activity score with CRP (DAS28-CRP), and **f** clinical disease activity index (CDAI). *ABT* abatacept, *TCZ* tocilizumab, *ETN* etanercept. *P < 0.05 tocilizumab vs. abatacept. **P < 0.01 tocilizumab vs. abatacept. †*P* < 0.05 etanercept vs. abatacept. ††*P* < 0.01 etanercept vs. abatacept

**Fig. 3:**
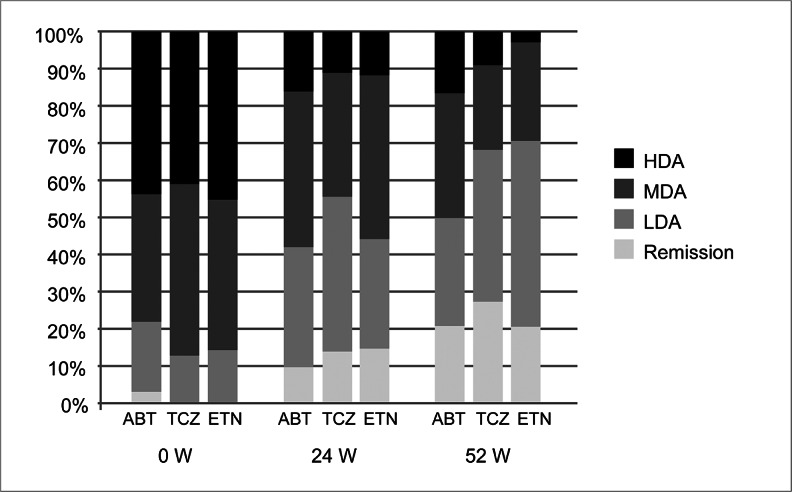
Clinical disease activity index (CDAI) with second-course biologics (0, 24, and 52 weeks). *ABT* abatacept, *TCZ* tocilizumab, *ETN* etanercept, *HDA* high disease activity (CDAI>22), *MDA* moderate disease activity (10<CDAI≤22), *LDA* low disease activity (CDAI≤10). Remission (CDAI≤2.8)

Figure 2 shows changes in tender joint counts, swollen joint counts, GH-VAS, CRP, DAS28-CRP, and CDAI at 0, 24, and 52 weeks. The decline over time in TJC, SJC, GH-VAS, CRP, DAS28-CRP, and CDAI significantly improved at all time points. TJC and SJC showed similar improvements without significant differences among the three drugs. GH-VAS was clearly higher in abatacept-treated patients (44.2 ± 27.3) compared to others (tocilizumab, 23.9 ± 23.0, *p* = 0.004; etanercept, 24.8 ± 20.8, *p* = 0.007) at 24 weeks, but there was no significant difference at 52 weeks. GH-VAS decreased more gradually in abatacept-treated patients. CRP levels were clearly lower with tocilizumab compared to abatacept at 24 weeks (tocilizumab, 0.16 ± 0.85; abatacept, 0.87 ± 1.16; *p* = 0.002) and 52 weeks (tocilizumab, 0.21 ± 0.87; abatacept, 0.91 ± 0.98; *p* = 0.001). DAS28-CRP showed no difference among the three drugs at 24 weeks but was lower with tocilizumab compared to abatacept at 52 weeks (tocilizumab, 2.51 ± 1.12; abatacept, 3.22 ± 1.11; *p* = 0.016). As shown in Fig. 3, all three drugs demonstrated good efficacy at 52 weeks in the evaluation based on CDAI. Remission rates and percentages of subsequent low disease activity for abatacept, tocilizumab, and etanercept were 20.7, 28.6, and 20.6 %, respectively, and 49.8, 68.2, and 70.6 %, respectively.

### Multivariate analysis

We calculated HRs for cause-specific drug discontinuation using multivariate Cox proportional HR analysis (Table 2) adjusted by disease duration, age, sex, concomitant MTX use, and CDAI. Discontinuation due to all unfavorable causes did not significantly differ among abatacept, tocilizumab, and etanercept, although discontinuation of tocilizumab due to adverse events and discontinuation of etanercept due to inadequate efficacy tended to be less common. There was no significant difference in inadequate efficacy and adverse events across the three drugs.

**Table 2:** Hazard ratios for discontinuation of the three drugs due to specific causes

	Etanercept	Tocilizumab	Abatacept
	(reference)	HR (95 % CI)	HR (95 % CI)
All unfavorable causes	1	0.58 (0.13–2.66)	1.21 (0.33–4.51)
Inadequate efficacy	1	1.37 (0.12–15.29)	2.14 (0.21–21.57)
Adverse events	1	0.28 (0.27–2.82)	0.77 (0.13–4.42)

Adjusted by sex, age, concomitant use of methotrexate, disease duration, and clinical disease activity index

*HR* hazard ratio, *CI* confidence interval

## Discussion

The recent introduction of two new biologics, abatacept and tocilizumab, into the market represents interesting new therapeutic opportunities for patients with RA who are resistant to TNFi. In the present study, no apparent difference in terms of efficacy was observed among abatacept, tocilizumab, and etanercept after switching from anti-TNF monoclonal antibodies.

In general, when patients respond poorly to the first TNFi after 3 to 4 months, switching to a different biologic agent is considered [6]. If the secondary loss of efficacy is due to anti-drug antibodies, switching to a second TNFi might prove effective [16]. In many cases, the first treatment is discontinued due to immunogenicity-related problems associated with the concomitant use of low-dose MTX. In such cases, the biologics with low immunogenicity are useful. Etanercept does not require concomitant MTX necessarily and could thus demonstrate the expected efficacy [2]. In contrast, if the secondary loss of efficacy is due to TNF no longer being the primary cytokine, switching to other classes of biologics will be required. Whenever possible, the switching of biologics should be decided based on the cause of secondary loss of efficacy; however, there is currently no method to determine this. Moreover, there is no consensus regarding the strategy of switching biologics.

The present observational study was based on data from a multicenter registry regarding the clinical efficacy of abatacept, tocilizumab, and etanercept in patients with RA in whom anti-TNF monoclonal antibody therapy previously failed. Therefore, the present results reflect treatment outcomes of the “real world.”

Several studies have reported on switching from TNFi to other biologics. One meta-analysis revealed no difference in ACR50 response to rituximab, tocilizumab, abatacept, and golimumab when switched from TNFi [15]. According to the Danish DANBIO study, 48-week retention rates of abatacept and tocilizumab after switching from TNFi were 54 and 64 %, respectively [7]. The retention rates in our study were better (68.0 % for abatacept and 89.5 % for tocilizumab). In the DANBIO study, the mean DAS28-CRP at 48 weeks was 3.3 for abatacept and 2.5 for tocilizumab, which were comparable to our results at 52 weeks (abatacept, 3.22 ± 1.11; tocilizumab, 2.51 ± 1.12). In addition, 48-week remission rate in the DANBIO study was 26 % for abatacept and 58 % for tocilizumab, which are better or almost the same as our results (17.4 and 55.6 %, respectively). The ATTAIN study, which examined patients who switched from TNFi to abatacept, reported the percentages of low disease activity and remission to be 24.2 and 13.9 %, respectively [25]. Compared to these, the percentages of low disease activity and remission in the present study were better (34.8 and 17.4 %, respectively). In the RADIATE study, DAS28 remission rate at 24 weeks (DAS28-CRP˂2.6) was 30.1 % in patients who switched from TNFi to tocilizumab [20], compared to 50.0 % in the present study. This difference might be attributed to low DAS28CRP values at baseline and the short disease duration of 7.9 ± 6.1 years in our study. As for patients who switched to etanercept from TNFi, the RADIUS study [26] reported a 52-week retention rate of 74 % in comparison to 84.6 % in our study. Taken together, our results are in good agreement with previous reports.

It should be emphasized that, in the present study, response rates and survival could not be compared among abatacept, tocilizumab, and etanercept due to the non-randomized, retrospective design. However, slight differences in clinical responses and disease activity (as judged by DAS28 CRP) among the three drugs appeared to be primarily due to the large decrease of CRP and ESR in tocilizumab-treated patients. Given that tocilizumab is an IL-6 antagonist and since IL-6 enhances the formation of CRP and ESR, our findings raise the question as to whether DAS28 is a valid tool for assessing disease activity for drugs that affect CRP and ESR. When evaluating tocilizumab, we believe that CDAI would serve as a useful tool since it does not involve CRP and ESR. In the present study, significant differences were found in CRP and DAS28-CRP when abatacept and tocilizumab were compared; however, as shown in Fig. 2f, there was no significant difference among the three drugs in terms of CDAI. The efficacy of the three drugs was found to be similar in the evaluation without CRP. In addition, the efficacy of tocilizumab was unchanged when the effects of CRP negativity was excluded.

In the present study, the three drugs showed no difference in therapeutic effects in patients with inadequate responses to anti-TNF monoclonal antibodies. In other words, abatacept and tocilizumab, which were found to be effective when switched from an anti-TNF monoclonal antibody, would offer good therapeutic options, as would etanercept in these patients. These biologics should be selected based on consultation with the patient regarding the method of administration (intravenous/subcutaneous injection) and dosing interval.

Limitations of this study include the small number of patients treated with each biologic agent. In the present study, it was necessary to set the study period after September 2010 as this was the year when abatacept was released in Japan. In addition, the number of patients requiring switching of medications was low since the long-term efficacy and safety of anti-TNF agents had been established. Nonetheless, use of the TBCR with over 2,000 cases enabled us to collect data for the present study. Given that the sample size might be insufficient to obtain strong statistical power, further studies will be necessary to reach the definite conclusion, yet our findings suggest no major differences among the three classes of biological DMARDs in terms of clinical efficacy after failure of first-course anti-TNF monoclonal antibody treatment. Additionally, given the retrospective design of the present study, drug selection was not randomized. Because of the bias of attending physicians, the number of cases that switched between anti-TNF monoclonal antibodies was quite low. As such, we were unable to evaluate in detail the switching between TNFi. Further evaluation is required for those who switch from anti-TNF monoclonal antibodies to another anti-TNF monoclonal antibody. Another limitation was the lack of data regarding the impact on structural damage (i.e., radiographic progression). These points should be addressed in the future.

In summary, we conclude that patients treated with either abatacept, tocilizumab, or etanercept can achieve a high response rate and that these biologics represent good therapeutic options in patients with RA who are refractory to first-course anti-TNF monoclonal antibody therapy. Moreover, the three biologics showed no significant difference in retention rate and efficacy. Further investigation to compare second-course anti-TNF monoclonal antibodies with the three drugs is needed to promote efficient drug selection when patients are switched from anti-TNF monoclonal antibodies.
